# Association between the glucose-to-lymphocyte ratio and prognosis in COVID-19 patients

**DOI:** 10.3389/fcimb.2026.1881669

**Published:** 2026-09-24

**Authors:** Cong Shi, Shengping Gong

**Affiliations:** 1 Stem Cell Laboratory, The First Affiliated Hospital of Ningbo University, Ningbo, Zhejiang, China; 2 Cancer Radiotherapy and Chemotherapy Center, The First Affiliated Hospital of Ningbo University, Ningbo, Zhejiang, China

**Keywords:** COVID-19, glucose-to-lymphocyte ratio, hospitalized, mortality, prognosis

## Abstract

**Background:**

The coronavirus disease 2019 (COVID-19) pandemic has transitioned into an endemic stage and is no longer classified as a public health emergency of international concern. Nevertheless, the persistent emergence of novel variants with enhanced transmissibility and immune evasion potential suggests that the disease burden associated with COVID-19 remains substantial. This retrospective cohort enrolled 235 hospitalized COVID-19 patients infected during the Omicron BA.5 and BF.7 wave from December 29, 2022 to January 15, 2023. The prognostic value of biomarkers based on combined fasting glucose and lymphocyte counts in patients with COVID-19 has not been fully clarified. We measured inflammatory markers, the metabolic marker, and T-cell immune parameters to validate our *a priori* hypothesis that glucose-to-lymphocyte ratio (GLR) mirrors concurrent inflammation. This study aimed to explore the associations between GLR and disease severity as well as clinical outcomes among hospitalized COVID-19 patients.

**Methods:**

We retrospectively analyzed a cohort of 235 individuals with COVID-19 to explore the prognostic value of GLR levels at diagnosis. All patients were followed up until the unified cutoff date of February 18, 2023, with all-cause mortality set as the primary endpoint. Two separate grouping methods were used: GLR stratification for evaluating its independent predictive ability, and severity grouping (mild-to-moderate vs severe) as clinical reference. The two groups only partially overlapped, so high GLR is not equivalent to severe illness. Patients were stratified into high GLR and low GLR groups. Overall survival (OS) was estimated via the Kaplan-Meier method and compared using the log-rank test.

**Results:**

Our findings revealed that GLR levels were significantly higher in severe patients than in mild-to-moderate patients. Meanwhile, beta2-microglobulin (β2-MG), neutrophil-to-lymphocyte ratio (NLR), platelet-to-lymphocyte ratio (PLR), and interleukin-10 (IL-10) were significantly elevated in the high GLR group, whereas apolipoprotein A1 (ApoA1), CD3 absolute value, CD4 absolute value, and CD8 absolute value were significantly lower. Furthermore, patients with high GLR levels exhibited significantly shorter OS (*P* < 0.0001). An elevated GLR level (> 14.42) was found to be an independent, adverse prognostic factor for OS in Omicron patients, according to multivariate Cox proportional hazards regression analysis (*P* = 0.003).

**Conclusions:**

Our data confirmed that GLR levels at initial diagnosis were significantly correlated with disease severity and prognosis in patients with COVID-19. As an easily calculated metabolic-immune indicator, GLR acts as a promising complementary candidate biomarker for mortality risk stratification in Omicron COVID-19 patients; further independent external validation is required before its routine clinical application.

## Introduction

Severe acute respiratory syndrome coronavirus 2 (SARS-CoV-2) infection, which is responsible for coronavirus disease 2019 (COVID-19), remains a global pandemic. Until now, the majority of COVID-19 related research has focused on the virus itself. Between December 29, 2022 and January 15, 2023, an outbreak caused by the Omicron BA.5 and BF.7 variants occurred in Ningbo, China. Medical institutions established specialized COVID-19 treatment centers specifically for patients infected with the Omicron variant, and these patients received standardized and systematic treatment. Omicron BA.5 and BF.7 subvariants differ markedly from wild-type SARS-CoV-2 and Delta variants in transmissibility, intrinsic virulence, and immune evasion capacity. Extensive mutations in the spike protein allow BA.5 and BF.7 to evade humoral immunity elicited by vaccination or prior infection. Their preferential replication within the upper respiratory tract attenuates pulmonary pathogenicity and remodels peripheral inflammatory and immune profiles. Such shifts in host response patterns may alter the predictive performance of hematological prognostic biomarkers (Zaderer et al., 2023).

Infection with the Omicron variant elicits a sequential cascade of immunological responses, covering both cellular and humoral immune pathways (Guo et al., 2026). Multiple biomarkers and risk factors have been identified that are closely linked to the development of severe COVID-19, such as peripheral blood absolute lymphocyte count (ALC), neutrophil-lymphocyte ratio (NLR), platelet-lymphocyte ratio (PLR), procalcitonin (PCT), D-dimer, Ferritin, and lactate dehydrogenase (Chan and Rout, 2020; Li et al., 2020; Liu et al., 2020; Qu et al., 2020; Lee et al., 2021; Elkhalifa, 2022; Martha et al., 2022). Our group has identified several risk factors linked to the development of severe COVID-19, including beta2-microglobulin (β2-MG), apolipoprotein E (ApoE), and apolipoprotein A1 (ApoA1) (Gong et al., 2023; Gong et al., 2024; Shi et al., 2025).

Glucose-to-lymphocyte ratio (GLR) refers to the ratio of fasting blood glucose to lymphocyte count. By integrating blood glucose levels with lymphocyte count, GLR provides a more comprehensive reflection of the body’s overall immune function (Zhou et al., 2025). Persistent hyperglycemia triggers oxidative stress and robust secretion of tumor necrosis factor-α (TNF-α), interleukin-6 (IL-6), and interleukin-1β (IL-1β). These pro-inflammatory cytokines suppress lymphocyte proliferation, induce T-cell apoptosis, and disrupt CD4^+^/CD8^+^ T-cell homeostasis. In addition, hyperglycemia activates the NF-κB signaling cascade, amplifying systemic inflammatory responses and exacerbating immune dysfunction in patients with COVID-19 (Drucker, 2021). Isolated blood glucose measurement only reflects glycemic dysregulation without capturing immune impairment, while absolute lymphocyte counts merely mirror immune status while overlooking metabolic injury. GLR normalizes lymphocyte counts by glycemic load; this single integrated metric simultaneously quantifies both metabolic and immune abnormalities, enabling more comprehensive prognostic evaluation (Wu et al., 2025). An elevated GLR indicates concomitant hyperglycemia and lymphopenia. Hyperglycemia-induced inflammatory activation combined with lymphocyte depletion synergistically attenuates host antiviral immunity, accelerates viral replication, aggravates pulmonary inflammatory lesions, and increases the risks of respiratory failure and in-hospital mortality. The metabolic-immune combined injury axis mediated by oxidative stress, NF-κB pathway activation, and pro-inflammatory cytokine release contributes to unfavorable clinical outcomes (Codo et al., 2020).

Increased GLR levels indicate abnormal glucose metabolism and immune function. While one machine learning analysis from Brazil has validated GLR as an early biomarker for severe COVID-19 (Ramalho-Pinto et al., 2025), few studies have clarified its prognostic value with respect to overall survival among COVID-19 hospitalizations. The prognostic value of GLR levels for overall survival in COVID-19 patients has not been fully elucidated. We therefore proposed the primary research hypothesis: COVID-19 patients presenting with higher GLR upon initial diagnosis exhibit poorer clinical outcomes and shorter survival.

## Materials and methods

### Patients

We conducted a retrospective study involving a cohort of 235 individuals diagnosed with COVID-19 via nucleic acid testing. All patients in this cohort were treated at the First Affiliated Hospital of Ningbo University from December 29, 2022 to January 15, 2023. All patients were tracked until a unified follow-up cutoff date of February 18, 2023, and all-cause mortality was defined as the primary study endpoint. Specifically, this group consisted of individuals experiencing their first episode of COVID-19 who had not received any prior symptomatic treatment. In order to confirm the presence of SARS-CoV-2 infection among all participants, viral nucleic acid testing was conducted, which required positive results for SARS-CoV-2 RNA. In accordance with the guidelines outlined in the “Protocol for the Diagnosis and Treatment of COVID-19 Patients (Tentative 8^th^ Edition),” participants were classified into two groups based on the severity of their condition: mild-to-moderate and severe (National Health Commission and the National Administration of Traditional Chinese Medicine, 2021). Mild-to-moderate COVID-19: Mild cases presented mild symptoms without pulmonary lesions on chest imaging; moderate cases showed fever and respiratory symptoms with visible pneumonia on radiology. Severe COVID-19 (adults, any single criterion met) (1) Respiratory rate ≥30 breaths/min; (2) Resting room-air SpO_2_ ≤93%; (3) PaO_2_/FiO_2_ ≤300 mmHg (adjusted for altitudes >1000 m); (4) Over 50% progression of lung lesions on imaging within 24–48 hours.

All patients hospitalized at our center during the study period received stratified, tiered treatment consisting of symptomatic supportive care, antiviral agents, anti-inflammatory therapy, and graded respiratory support. Glucocorticoids were reserved exclusively for severe and critically ill patients. Mild-to-moderate cases received nasal cannula or face mask oxygen supplementation, whereas non-invasive or invasive mechanical ventilation was implemented for severe patients. Antiviral treatments including lopinavir/ritonavir and ribavirin were administered to individuals with severe disease.

The Ethics Committee waived dedicated study-specific informed consent for this anonymized retrospective observational analysis, as no additional clinical intervention or direct participant contact was performed. Following the guidelines set forth in the Declaration of Helsinki, this study was designed as a single-center retrospective analysis and approved by the Ethics Committee of the First Affiliated Hospital of Ningbo University (Approval No.: 2026020A). The study was reported in strict accordance with the STROBE (Strengthening the Reporting of Observational Studies) guidelines for observational studies.

### Inclusion and exclusion criteria

Patients were included in the study based on specific criteria to ensure homogeneity and relevance to the research objectives. Patients were included if they met all of the following criteria: diagnosed with COVID-19 via SARS-CoV-2 real-time quantitative polymerase chain reaction (PCR) nucleic acid testing; identified as having BA.5 or BF.7 Omicron subvariant infection using the Oxford Nanopore third-generation whole-genome sequencing platform; received inpatient treatment, completed complete blood count and biochemical laboratory examinations; had comprehensive clinical data available for analysis, and were aged 18 years or older. Patients with a prior history of COVID-19 infection were excluded. Ultimately, 235 participants met the inclusion criteria (**Figure 1**).

**Figure 1:**
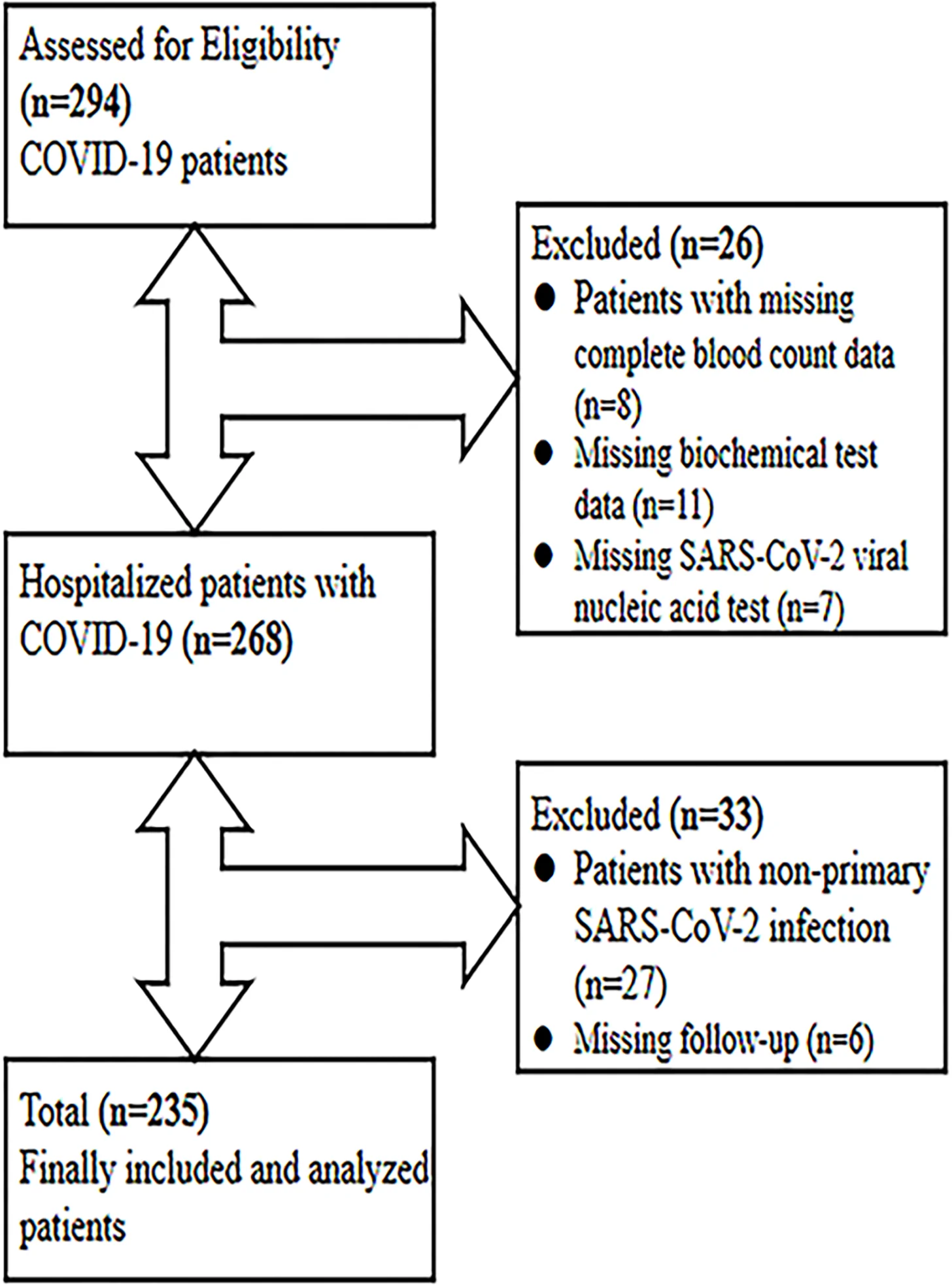
Flowchart of the study design and patient data enrolment.

### Clinical characteristics and laboratory data

Epidemiological characteristics, including recent exposure history, clinical symptoms and signs, as well as laboratory findings, were collected from electronic medical records and verified by telephone follow-up. Laboratory examinations included complete blood count, blood biochemistry, CRP, cytokine levels, and absolute counts of lymphocyte subsets. Peripheral blood samples from all participants were anticoagulated with ethylenediaminetetraacetic acid (EDTA). Complete blood count and CRP were assayed on a Sysmex XN-10 automated hematology analyzer with matched proprietary reagents: complete blood count was measured by flow cytometry with fluorescent staining, and CRP via nephelometric turbidimetry. Lymphocyte subsets and cytokines were analyzed on a BD FACSCanto flow cytometer to obtain absolute counts. Cytokines were detected using the BD Human Th1/Th2 Cytokine Kit, and lymphocyte subsets were quantified with BD Multitest 6-Color TBNK Reagent. All patients fasted for at least 6 hours before blood collection. Biochemical parameters, including fasting glucose measured by the hexokinase method, were tested on a Beckman AU5800 automatic biochemistry analyzer with supporting kits. All assays were performed strictly following manufacturer’s protocols. GLR was defined as the ratio of fasting glucose to lymphocyte count (Navarro et al., 2019), and mortality was set as the primary outcome of this study.

### Statistical analysis

All statistical analyses in this study were performed using SPSS 31.0, R software 4.4.1, and GraphPad Prism 8.0. For survival analysis, Overall survival was defined as the time from the COVID-19 hospital diagnosis to the follow-up cut-off date of February 18, 2023. Group differences in OS were compared using the Kaplan–Meier survival method with the log-rank test, and independent prognostic factors were further explored via multivariate Cox proportional hazards regression analysis.

Prior to pairwise comparison, the Shapiro–Wilk test was applied to assess the normality of all continuous variables, and all continuous indicators were confirmed to follow a non-normal distribution. Accordingly, intergroup differences were statistically analyzed using appropriate methods matched to variable types: the chi-squared test for categorical variables and the Mann–Whitney U test for non-normally distributed continuous variables.

Considering that standardized laboratory reference values for the GLR in predicting COVID-19 patient survival have not been established and relevant clinical research remains limited, we performed receiver operating characteristic (ROC) curve analysis to evaluate the prognostic efficacy of GLR. The optimal GLR cut-off value was determined by maximizing the Youden index combined with maximally selected rank statistics.

A comprehensive set of indicators was included for statistical screening in this study, covering blood routine parameters, conventional infection monitoring indicators, lymphocyte subset counts, and previously reported COVID-19 prognostic biomarkers identified by our team (including β2-microglobulin and apolipoprotein A1). All enrolled indicators were compared between high and low GLR subgroups. Specifically, univariate analysis was first conducted to screen potential prognostic indicators, and variables with a univariate *P* < 0.05 were subsequently incorporated into the multivariate Cox regression model for further analysis.

Strict model validity verification was performed to ensure the reliability of the Cox regression results. The Schoenfeld residual test was used to verify the proportional hazards assumption, and Pearson correlation analysis between Schoenfeld residuals and rank-ordered survival time showed no significant time-dependent effects for all covariates (all *P* > 0.05), confirming compliance with the proportional hazards assumption. Martingale residuals and deviance residuals were used for graphical goodness-of-fit assessment, and visual inspection of residual plots showed no systematic deviation or obvious nonlinear trends, indicating a good model fit. Score residuals (DiBeta statistics) were adopted to screen influential observations, and no extreme outlier cases were detected. Furthermore, sensitivity analysis by excluding suspected outliers yielded stable hazard ratio results with no substantial fluctuations, verifying the robustness of our statistical results.

A *P* value < 0.05 was defined as the threshold of statistical significance for all analyses.

## Results

### Baseline characteristics

A total of 235 patients were included in the analysis. Maximally selected rank statistics for identifying the optimal cut-off for survival stratification and Youden index-based ROC analysis both produced the optimal GLR cut-off value of 14.42. Accordingly, participants were divided into a high GLR group (49%) and a low GLR group (51%). The median age of the study population was 76 years, and 64% of patients were male. All patients were followed until February 18, 2023. Within this population, the median overall survival time was found to be 44 days, with a range spanning from 2 to 63 days, during which 61 fatalities were reported. Compared with the low GLR group, patients in the high GLR group exhibited significantly elevated levels of β2-MG (*P* = 0.028), NLR (*P <* 0.001), and PLR (*P <* 0.001). In addition, the high GLR group had lower ApoA1 (*P* = 0.008) and ALC (*P* < 0.001), as well as fewer absolute counts of CD3, CD4, CD8, NK, and CD19 lymphocytes compared with the low GLR group (all *P* < 0.001). No significant differences were observed in other variables between the two groups. Detailed characteristics are summarized in **Table 1**. GLR levels were significantly higher in severe patients than in mild-to-moderate patients. Similarly, non-surviving patients had markedly higher GLR levels than surviving patients (all *P* < 0.001) (**Figures 2A, B**).

**Table 1:** Comparison between omicron with low GLR group and high GLR group in 235 patients.

Variable	Reference ranges	Low GLR group (*n* = 120)	High GLR group (n=115)	Statistics	*P-value*
Male/Female, n	–	71/49	79/36	χ2 = 2.310	0.129
Age **[**years, median(quartile)**]**	–	74 (66~84)	78 (69~85)	Z=-1.484	0.138
Comorbidity, n					
Hypertension	–	68/52	75/40	χ2 = 1.802	0.179
Diabetes	–	24/96	40/75	χ2 = 6.475	0.011
Cardiovascular disease	–	8/112	5/110	χ2 = 0.604	0.437
Laboratory parameters					
WBC [×10^9^/L, median(quartile)]	3.5-9.5	7.2 (5.0~10.5)	7.2 (4.3~11.1)	Z=-0.473	0.662
NE [×10^9^/L, median(quartile)]	1.8-6.3	5.6 (3.3~8.1)	6.6 (3.5~10.2)	Z=-1.506	0.132
ALC [×10^9^/L, median(quartile)]	1.1-3.2	0.9 (0.7~1.3)	0.4 (0.3~0.5)	Z=-11.061	<0.001
RBC [×10^12^/L, median(quartile)]	4.3-5.8	3.9 (3.4~4.3)	3.9 (3.4~4.4)	Z=-0.200	0.842
HB [g/L, median(quartile)]	130-175	121 (109~133)	119 (106~137)	Z=-0.061	0.951
PLT [×10^9^/L, median(quartile)]	125-350	185 (149~248)	184 (123~247)	Z=-1.370	0.171
CRP [mg/L, median(quartile)]	0.5-10	40 (16.4~81.3)	49.2 (21.4~101.2)	Z=-1.798	0.072
β2-MG [mg/L, median(quartile)]	1-2.3	3.2 (2.2~5.0)	3.6 (2.5~6.7)	Z=-2.192	0.028
ApoE [mg/L, median(quartile)]	29-53	46.4 (38.8~60.3)	49.8 (39.9~64.7)	Z=-1.599	0.110
ApoA1 [g/L, median(quartile)]	0.8-1.6	0.9 (0.8~1.0)	0.8 (0.7~1.0)	Z=-2.648	0.008
NLR [median(quartile)]	–	5.9 (3.1~9.7)	14.4 (7.8~27.5)	Z=-7.600	<0.001
PLR [median(quartile)]	–	210 (136~292)	410 (273~605)	Z=-8.323	<0.001
GLR [median(quartile)]	–	9.1 (5.3~11.8)	24.4 (18.4~35.7)	Z=-13.245	<0.001
Cytokines					
IL-6 [pg/ml, median(quartile)]	0-6.28	21.2 (12.0~59.4)	22.6 (11.0~72.1)	Z=-0.546	0.585
IL-10 [pg/ml, median(quartile)]	0-6.09	9.7 (6.2~15.5)	10.7 (7.1~23.7)	Z=-1.788	0.074
Lymphocyte subpopulation					
CD3 absolute value [/μl, median(quartile)]	797-2370	427 (277~727)	176 (122~245)	Z=-7.815	<0.001
CD4 absolute value [/μl, median(quartile)]	432-1341	244 (143~422)	103 (69~147)	Z=-7.041	<0.001
CD8 absolute value [/μl, median(quartile)]	238-1075	150 (91~275)	59 (32~102)	Z=-6.439	<0.001
NK absolute value [/μl, median(quartile)]	127-987	136 (81~210)	85 (46~147)	Z=-3.935	<0.001
CD19 absolute value [/μl, median(quartile)]	86-594	96 (48~164)	53 (23.5~86.5)	Z=-4.827	<0.001

WBC, white blood count; NE, neutrophil; ALC, absolute lymphocyte count; RBC, red blood count; HB, hemoglobin; CRP, C reactive protein; β2-MG, beta-2 microglobulin; ApoE, apolipoprotein E; ApoA1, apolipoprotein A1; NLR, neutrophil-to-lymphocyte ratio; PLR, platelet-to-lymphocyte ratio; GLR, glucose-to-lymphocyte ratio; IL-6, interleukin-6; IL-10, interleukin-10.

**Figure 2:**
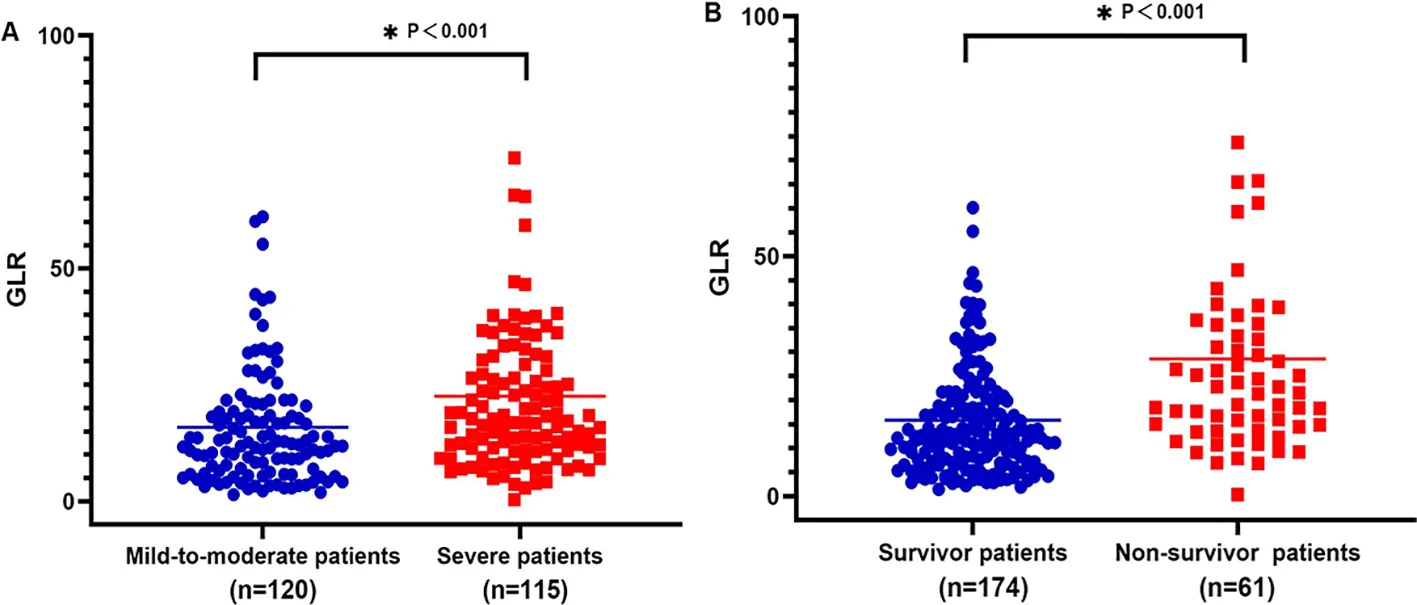
**(A)** Compare GLR between 120 mild-to-moderate patients and 115 severe patients. **(B)** Compare GLR between 174 survivor patients and 61 non-survivor patients. *P <0.001.

### Use of the optimum cut-off value of GLR to discern survival from non-survival COVID-19 infection

We determined the optimal cut-off value using ROC analysis, with the ROC curves presented in **Figure 3**. As illustrated in **Figure 3**, the areas under the curve (AUC) for GLR, NLR, and PLR were 0.715, 0.697, and 0.615, respectively. Pairwise DeLong test was performed to compare the AUCs of the three indicators. As shown in the heatmap, the AUC of GLR was significantly higher than that of PLR (*P* = 0.003), and the AUC of NLR was also significantly superior to PLR (*P* = 0.015). No statistically significant difference was observed between the AUCs of GLR and NLR (*P* = 0.615). The optimal cut-off value of GLR was 14.42, with a sensitivity of 75.4% and a specificity of 60.3% (see **Table 2**). GLR showed moderate discriminative capacity. Although it had the numerically largest AUC among the three markers, its predictive performance was statistically superior only to PLR, with no significant difference detected when compared with NLR.

**Figure 3:**
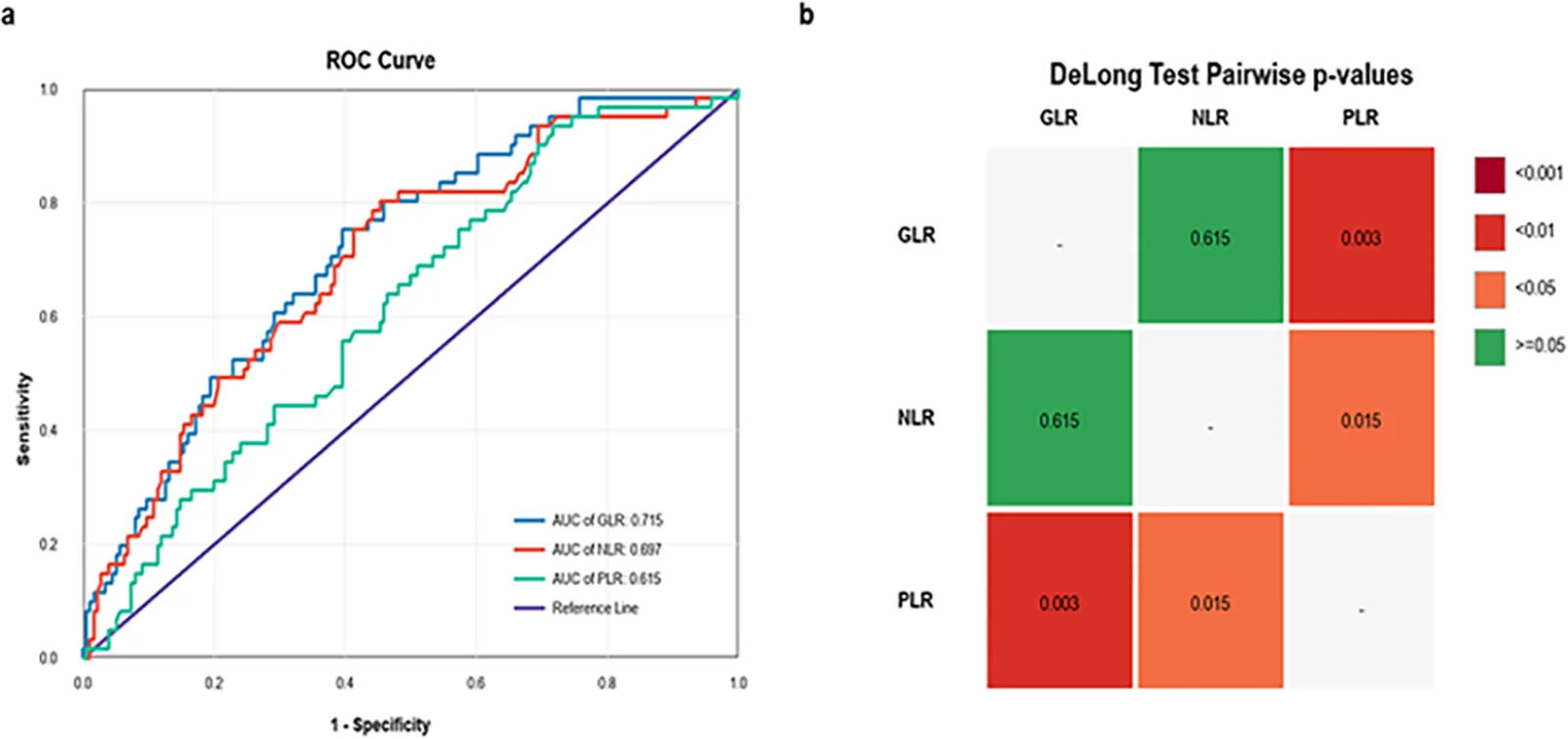
ROC curve analysis and pairwise DeLong test comparisons of GLR, NLR, and PLR. **(a)** ROC curves of GLR, NLR, and PLR. **(b)** Heatmap displaying pairwise *P* values derived from the DeLong test.

**Table 2:** Areas under the curve (AUC) of GLR, NLR, and PLR.

Variables	Area	Std. error^a^	Asymptotic Sig.^b^	Asymptotic 95% confidence interval	
				Lower bound	Upper bound
GLR	0.715	0.037	0.000	0.644	0.787
NLR	0.697	0.039	0.000	0.621	0.773
PLR	0.615	0.040	0.004	0.537	0.693

a Under the nonparametric assumption.

b Null hypothesis: true area = 0.5.

### Linear correlations between peripheral blood markers

A linear correlation model revealed an association between GLR and peripheral blood markers. Correlation analysis showed that GLR was positively correlated with β2-MG, NLR, PLR, and IL-10, but negatively correlated with ApoA1 and the absolute counts of CD3, CD4, and CD8. These significant correlations suggested that the combined indices could serve as better indicators for assessing disease severity (**Figure 4**).

**Figure 4:**
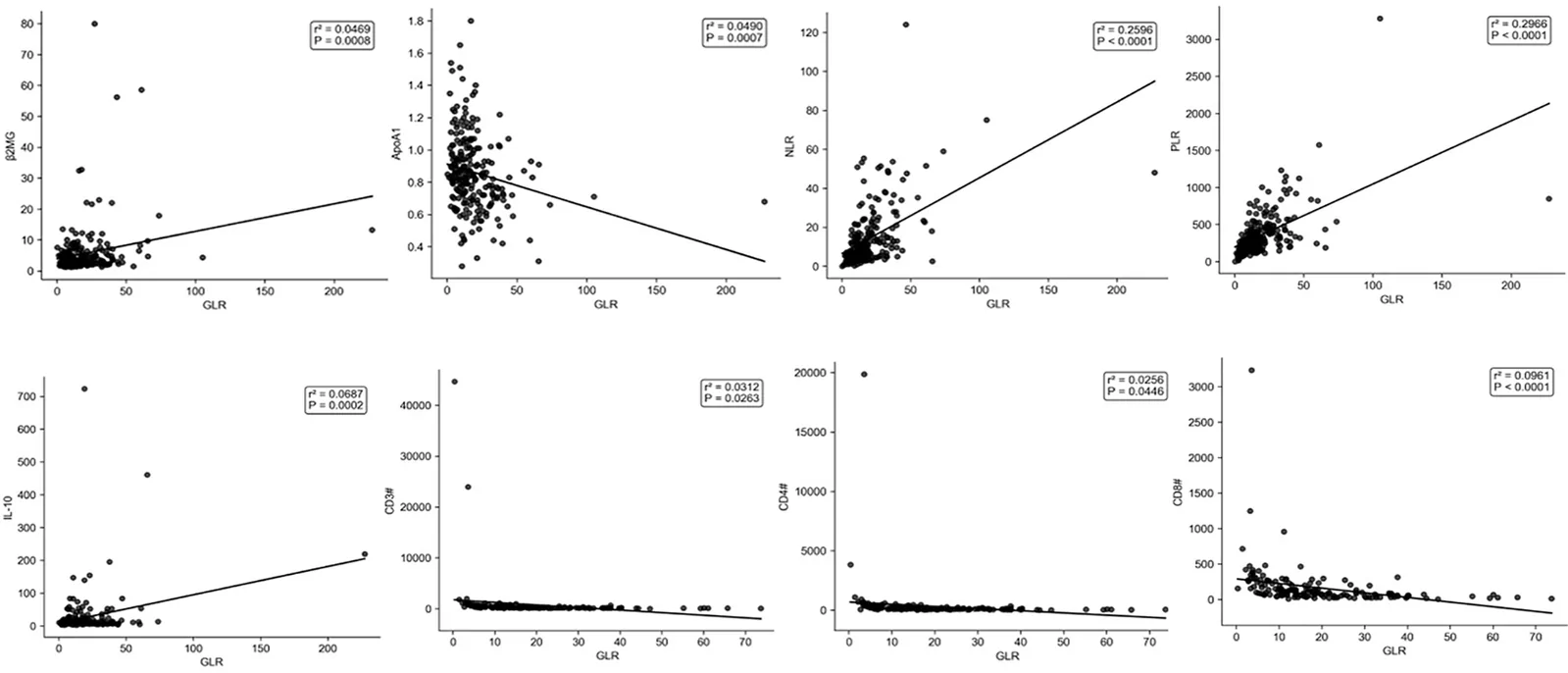
Linear correlations between the GLR and peripheral blood markers.

### High GLR level was associated with a poor prognosis

Patients with high GLR levels had a significantly shorter median OS than those with low GLR levels (*P* < 0.0001; **Figure 5**). Univariate analysis demonstrated that shorter OS was associated with disease severity (*P* < 0.001), elevated CRP, IL-6, IL-10, WBC, NE, β2-MG, and GLR (all *P* < 0.001), as well as reduced RBC (*P* = 0.035), HB (*P* = 0.004), PLT (*P* = 0.005), and ApoA1 (*P* < 0.001).

**Figure 5:**
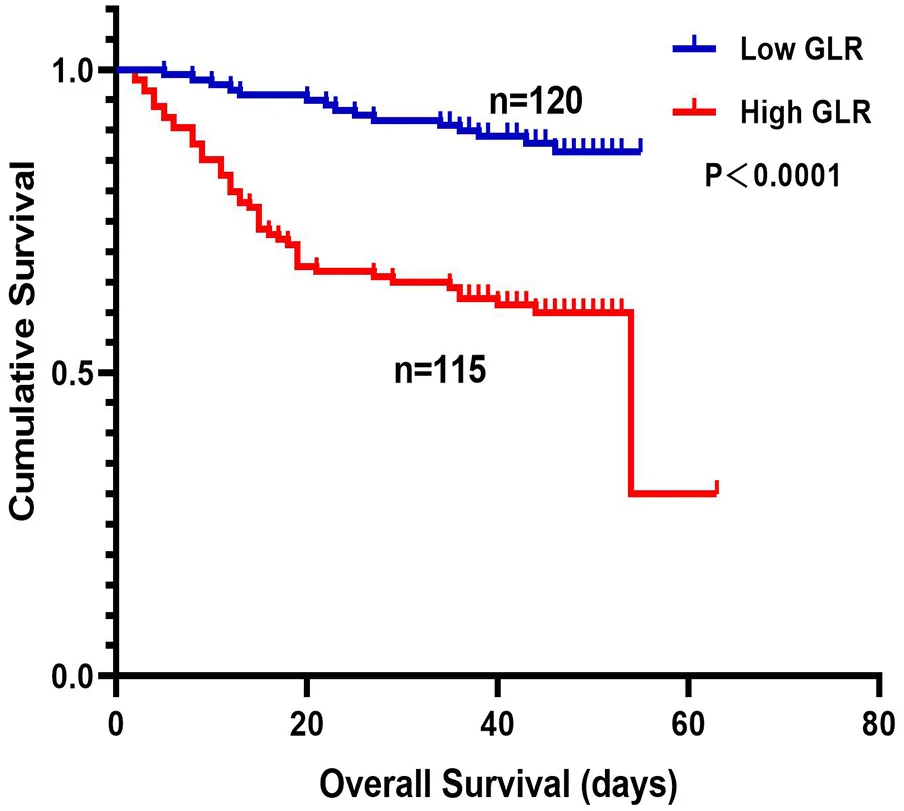
Overall survival according to GLR in COVID-19 patients.

To mitigate overfitting caused by the limited number of mortality events (n = 61), we constructed a clinically pre-specified parsimonious multivariate Cox model including only five biologically relevant covariates: severe patient status, β2-MG, ApoA1, IL-10, and GLR. This pre-defined five-variable primary model yielded an events-per-variable ratio of 12.2. Multivariate analysis based on this optimized model identified severe disease status (*P* < 0.001, *HR* = 7.783), elevated β2-MG (*P* < 0.001, *HR* = 3.045), and high GLR (*P* = 0.003, *HR* = 2.615) as independent adverse predictors of poor OS, while IL-10 (*P* = 0.054) and ApoA1 (*P* = 0.095) did not reach statistical significance in the adjusted model (**Table 3**). The original data-driven saturated multivariate model incorporating all univariate significant indicators was moved to **Supplementary Table 2** for exploratory reference only.

**Table 3:** Univariate and multivariate analyses of different prognostic parameters for overall survival of 235 patients.

Variables	Univariate analysis for OS		Multivariate analysis for OS		
	*P-value*	95%CI	*P-value*	*HR*	95%CI
IL-10 ≥ 28.3pg/ml	<0.001	27.220-40.092	0.054	1.808	0.990-3.302
Severe patients	<0.001	37.247-54.753	<0.001	7.783	2.719-22.277
β2-MG ≥ 8.89mg/L	<0.001	12.291-26.959	<0.001	3.045	1.664-5.573
ApoA1 < 0.68 g/L	<0.001	32.533-47.154	0.095	0.549	0.272-1.109
GLR > 14.42	<0.001	35.568-46.680	0.003	2.615	1.377-4.967

β2-MG, beta-2 microglobulin; ApoA1, apolipoprotein A1; IL-10, interleukin-10, GLR, glucose-to-lymphocyte ratio.

GLR showed no significant correlation with survival in mild-to-moderate patients (*P* > 0.05), which may be attributed to the limited number of death events (only 5 deaths) within this subgroup.

In severe COVID-19 patients, Kaplan-Meier survival analysis revealed that severe patients with elevated GLR exhibited markedly shortened median OS relative to individuals with low GLR (*P* < 0.001, **Figure 6**). Univariate survival regression identified several variables correlated with unfavorable OS, including higher IL-10 (*P* = 0.004), β2-MG (*P* < 0.001), GLR (*P* < 0.001), and decreased PLT (*P* = 0.001). Multivariate Cox regression further confirmed that raised β2-MG (*P* = 0.005) and GLR (*P* = 0.022) served as independent negative prognostic indicators (**Table 4**).

**Figure 6:**
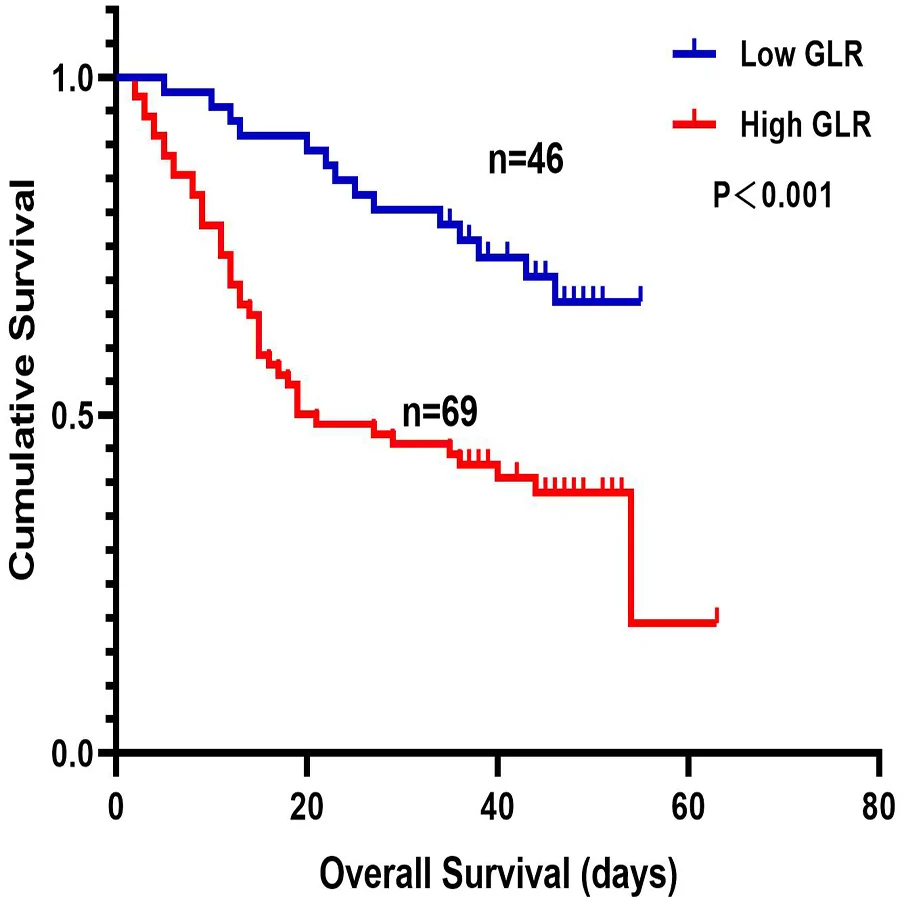
Overall survival according to GLR in severe COVID-19 patients.

**Table 4:** Univariate and multivariate analyses of different prognostic parameters for overall survival of 115 severe patients.

Variables	Univariate analysis for OS		Multivariate analysis for OS		
	*P-value*	95%CI	*P-value*	*HR*	95%CI
Age ≥ 60(years)	0.769	35.251-44.731	0.304	2.055	0.521-8.105
Gender (male)	0.945	35.364-45.821	0.602	1.226	0.569-2.643
CRP ≥ 52.23mg/L	0.096	30.006-41.849	0.209	0.617	0.290-1.311
IL-6 ≥ 38.7pg/ml	0.088	28.039-38.920	0.559	0.764	0.309-1.887
IL-10 ≥ 28.3pg/ml	0.004	20.115-33.105	0.142	1.919	0.804-4.580
WBC ≥ 14.9×10^9^/L	0.172	23.091-40.271	0.328	0.385	0.057-2.613
NE ≥ 13.3×10^9^/L	0.185	22.341-40.314	0.896	0.872	0.111-6.848
RBC < 3.57×10^9^/L	0.166	25.953-39.336	0.685	0.830	0.338-2.041
HB < 100g/L	0.068	20.515-39.202	0.185	2.040	0.711-5.854
PLT < 134×10^9^/L	0.001	20.410-34.282	0.160	1.877	0.779-4.520
Hypertension	0.486	35.837-47.031	0.118	1.749	0.868-3.527
Cardiopathy	0.414	4.574-44.092	0.258	0.265	0.026-2.650
Diabetes	0.686	28.792-45.151	0.723	1.142	0.549-2.377
Severe patients	–	–	–	–	–
β2-MG ≥ 8.89mg/L	<0.001	24.349-36.804	0.005	2.821	1.378-5.776
ApoA1 < 0.68 g/L	0.177	31.734-43.027	0.160	0.524	0.213-1.291
GLR > 14.42	<0.001	25.852-37.731	0.022	2.468	1.137-5.357

WBC, white blood count; NE, neutrophil; RBC, red blood count; HB, hemoglobin; CRP, C reactive protein; β2-MG, beta-2 microglobulin; ApoA1, apolipoprotein A1; IL-6, interleukin-6; IL-10, interleukin-10, GLR, glucose-to-lymphocyte ratio.

## Discussion

In the present study, admission elevated GLR was significantly associated with unfavorable survival among hospitalized COVID-19 patients and demonstrated satisfactory predictive performance for clinical outcomes. GLR exhibited synchronous dynamic trends with canonical inflammatory ratios (NLR and PLR), biomarkers reflecting perturbed immune-inflammatory homeostasis during disease progression. Rather than functioning as an entirely independent standalone marker, GLR serves as a complementary and synergistic parameter to conventional inflammatory indices. Incorporating GLR alongside NLR and PLR into biomarker panels enables integrated evaluation of systemic inflammatory burden and lymphocyte-mediated immune impairment, overcoming the insufficient predictive capacity of single indicators and supporting refined prognostic stratification. Notably, maximally selected rank statistics and Youden index-based ROC analysis yielded an identical GLR cut-off value of 14.42, reinforcing the robustness of this threshold for risk prediction. Mechanistically, the prognostic value of GLR hinges on the coexistence of hyperglycemia and lymphopenia in severe infection. As an easily accessible biomarker integrating metabolic and immune phenotypes, GLR shows preliminary value for auxiliary early risk stratification in hospitalized Omicron COVID-19 patients, yet external cohort validation is needed to confirm its generalizability before widespread clinical use.

Moreover, high GLR level is an independent risk factor for poor prognosis in COVID-19 patients, and this is particularly more obvious in severe cases compared to mild-to-moderate cases.

GLR has emerged as an economical inflammatory biomarker with broad pathological relevance and promising utility for quantifying systemic inflammatory responses. Prior investigations have validated GLR as a prognostic marker across multiple disorders, including solid malignancies (Liu et al., 2024), chronic obstructive pulmonary disease (Hu et al., 2022), and acute respiratory distress syndrome (Zhang and Zhang, 2022). Despite accumulating evidence supporting its clinical utility, few studies have systematically explored the prognostic implication of GLR in Omicron BA.5/BF.7 infection, highlighting the novelty of our present work.

Immunometabolic crosstalk is central to determining disease trajectories in COVID-19. Hyperglycemia suppresses lymphocyte function and amplifies inflammatory cascades, whereas lymphopenia represents a hallmark of severe COVID-19 and reflects compromised antiviral immunity. As a composite index, GLR simultaneously captures metabolic stress and cellular immune deficiency. In our cohort, GLR positively correlated with β2-MG, NLR, PLR and IL-10, while negatively correlating with ApoA1 and CD3^+^, CD4^+^, CD8^+^ lymphocyte counts. These correlation patterns corroborate that GLR encapsulates the combined phenotypes of metabolic disturbance, systemic inflammation and impaired cellular immunity. Conventional inflammatory indices NLR and PLR only mirror the balance of circulating leukocyte subsets (Islam et al., 2024). Distinctively, GLR integrates glycemic concentration and lymphocyte counts, enabling simultaneous assessment of two intertwined pathological features of COVID-19: metabolic derangement and immune insufficiency. Severe SARS-CoV-2 infection frequently triggers stress hyperglycemia, which further aggravates lymphocyte dysfunction. Therefore, GLR can capture combined metabolic and immune abnormalities that isolated leukocyte ratios fail to identify. Consistent with this mechanistic rationale, pairwise ROC comparisons in our analysis demonstrated that GLR yielded the numerically highest AUC value, though its predictive performance was not statistically superior to NLR and only outperformed PLR significantly. NLR and PLR merely reflect shifts in peripheral inflammatory cell populations and incompletely characterize the systemic inflammatory burden triggered by COVID-19. By contrast, GLR incorporates two independent pathophysiological readouts: blood glucose and absolute lymphocyte count. Hyperglycemia propagates inflammatory cascades and impairs cellular immunity, whereas lymphopenia signifies immune reserve exhaustion. By integrating these two core pathological alterations, GLR possesses greater discriminatory ability than single immune cell ratios. Practically, fasting glucose and lymphocyte counts are routinely tested upon admission, rendering GLR a convenient, non-invasive, low-cost candidate for early risk screening without additional laboratory tests. Nevertheless, several inherent caveats of GLR deserve consideration. Peripheral glucose levels are susceptible to acute stress, corticosteroid treatment and intravenous fluid infusion during hospitalization, which may induce transient GLR fluctuations. Unlike NLR and PLR derived purely from leukocyte subsets, GLR incorporates glycemic parameters and can be disturbed by non-infectious acute factors. Accordingly, GLR should be interpreted combined with complete clinical information instead of being used in isolation.

Multiple validated scoring systems, including NEWS2, CURB-65 and the 4C Mortality Score, are widely implemented to assess COVID-19 severity and mortality risk. NEWS2 depends on serial vital sign measurements to detect acute physiological deterioration but cannot capture chronic metabolic and immune disturbances underlying COVID-19. CURB-65 was developed for community-acquired bacterial pneumonia and lacks dedicated validation for Omicron variants, limiting its performance in our study population. Although the 4C Mortality Score achieves strong prognostic efficacy for COVID-19, its calculation requires numerous variables, hindering rapid bedside screening during admission. Compared with these scoring tools, GLR is calculated solely from routine admission laboratory parameters, without repeated vital sign monitoring or extensive clinical data collection, making it particularly suitable for busy emergency departments and wards. Importantly, we do not propose GLR as a replacement for established clinical risk scores; combining GLR with standardized scoring systems may further improve prognostic discrimination. Regrettably, complete variables required to compute NEWS2, CURB-65 and the 4C Mortality Score were unavailable for all enrolled patients in this single-center retrospective cohort, preventing formal quantitative head-to-head comparison of AUC and discriminatory performance between GLR and these scoring models. Multivariate Cox regression identified low ApoA1, elevated IL-10, elevated β2-MG and elevated GLR as independent predictors of inferior overall survival. These findings align with existing literature emphasizing the critical roles of immune activation and inflammatory injury in COVID-19 pathogenesis.

Several limitations of this study need to be acknowledged. First, the single-center retrospective design and moderate sample size may restrict the external generalizability of our conclusions. Second, only baseline admission GLR was analyzed; serial dynamic GLR changes throughout hospitalization were not evaluated. Third, the optimal cut-off value of 14.42 was derived exclusively from the present cohort, raising concerns regarding overfitting and multiple testing bias. External validation in independent cohorts is essential to verify this threshold. Fourth, GLR is calculated by GLU/ALC mathematical formulas. The mathematical dependency among the three variables results in a weakened predictive effect of GLR when all three variables are included in the model. Moreover, admission normoglycemia cannot exclude occult diabetes mellitus, a major confounding variable for analyses of inflammatory and metabolic phenotypes. Incomplete BMI recording further precluded comprehensive adjustment for obesity-related metabolic risk. Fifth, all participants were infected with Omicron BA.5/BF.7 during a single regional outbreak in Ningbo. Accordingly, our predictive model and GLR cut-off cannot be directly generalized to populations infected with other Omicron sublineages with divergent virological properties. Sixth, only 61 mortality events occurred in our cohort, yielding a low events-per-variable ratio and potential risk of model overfitting. To achieve stable hazard ratio estimation, we adopted strict variable filtering and excluded redundant predictors. We constructed a parsimonious primary model incorporating just five clinically relevant covariates (severe patient status, β2-MG, ApoA1, IL-10 and GLR), aiming to improve the reliability of our Cox regression results (**Supplementary Table 2**). Seventh, incomplete data for NEWS2, CURB-65 and 4C Mortality Score prohibited direct comparison of predictive performance between GLR and established prognostic scoring tools. Future prospective multicenter studies with complete scoring data are warranted to quantify the incremental prognostic value of GLR when combined with validated risk stratification frameworks. Finally, several unmeasured confounders remain unadjusted, such as the type and duration of immunomodulatory therapies, which may introduce residual confounding. Further large-scale prospective cohorts are required to corroborate the prognostic value of GLR among COVID-19 patients. These unresolved issues underline the necessity of future large prospective cohorts to systematically investigate the effects of standardized systemic glycemic control on disease outcomes in COVID-19 patients. Collectively, given all the above limitations, GLR should only be regarded as a complementary candidate biomarker rather than a mature, universally applicable prognostic tool, and multicenter prospective studies are warranted to verify its predictive efficiency in diverse patient populations.

In conclusion, elevated admission GLR is closely associated with greater disease severity and poor prognosis in hospitalized COVID-19 patients. Our results preliminarily indicate that elevated admission GLR correlates with severe disease and poor survival among hospitalized Omicron COVID-19 patients. As a readily available multi-dimensional marker reflecting combined metabolic and immune disturbance, GLR represents a promising complementary candidate for auxiliary risk stratification. Given the single-center retrospective design and absence of external validation, further independent cohort verification is required to confirm its clinical utility before it can be routinely applied to guide clinical decision-making.

## Data Availability

The original contributions presented in the study are included in the article/Supplementary Material. Further inquiries can be directed to the corresponding author.
