# Supplementary material for: Association between the glucose-to-lymphocyte ratio and prognosis in COVID-19 patients
Source: Front Cell Infect Microbiol. 2026 Sep 24;16:1881669. doi: 10.3389/fcimb.2026.1881669 (PMC13645524; doi:10.3389/fcimb.2026.1881669)
Supplement: Supplementary file 1 [file Table1.docx]

**Table S1. Univariate and multivariate analyses of different prognostic parameters for overall survival of 235 patients**

| **Variables** | **Univariate analysis for OS** | | **Multivariate analysis for OS** | | |
| --- | --- | --- | --- | --- | --- |
|  | ***P-value*** | **95%CI** | ***P-value*** | ***HR*** | **95%CI** |
| Age ≥ 60(years) | 0.279 | 47.279-53.222 | 0.391 | 1.817 | 0.464-7.112 |
| Gender (male) | 0.337 | 46.649-53.513 | 0.432 | 1.322 | 0.659-2.650 |
| CRP ≥ 52.23mg/L | <0.001 | 36.833-47.964 | 0.295 | 0.683 | 0.335-1.394 |
| IL-6 ≥ 38.7pg/ml | <0.001 | 26.422-41.605 | 0.720 | 0.855 | 0.363-2.015 |
| IL-10 ≥ 28.3pg/ml | <0.001 | 27.220-40.092 | 0.136 | 1.889 | 0.819-4.359 |
| WBC ≥ 14.9×10^9^/L | <0.001 | 26.359-42.650 | 0.301 | 0.359 | 0.051-2.501 |
| NE ≥ 13.3×10^9^/L | <0.001 | 24.798-42.128 | 0.994 | 0.992 | 0.126-7.830 |
| ALC < 0.7×10^9^/L | <0.001 | 40.725-49.418 | 0.310 | 1.601 | 0.646-3.970 |
| RBC < 3.57×10^9^/L | 0.035 | 36.628-45.501 | 0.554 | 0.770 | 0.323-1.833 |
| HB < 100g/L | 0.004 | 31.158-44.700 | 0.079 | 2.400 | 0.905-6.370 |
| PLT < 134×10^9^/L | 0.005 | 34.745-44.827 | 0.295 | 1.532 | 0.689-3.408 |
| Hypertension | 0.678 | 46.975-53.986 | 0.247 | 1.480 | 0.762-2.872 |
| Cardiopathy | 0.820 | 33.954-54.969 | 0.661 | 0.620 | 0.073-5.259 |
| Diabetes | 0.399 | 39.218-52.657 | 0.964 | 1.017 | 0.490-2.111 |
| Severe patients | <0.001 | 37.247-54.753 | <0.001 | 8.104 | 2.681-24.501 |
| β2-MG ≥ 8.89mg/L | <0.001 | 12.291-26.959 | 0.001 | 3.219 | 1.635-6.336 |
| ApoA1 < 0.68 g/L | <0.001 | 32.533-47.154 | 0.089 | 0.484 | 0.209-1.116 |
| GLR > 14.42 | <0.001 | 35.568-46.680 | 0.126 | 1.973 | 0.825-4.716 |

Abbreviations: WBC, white blood count; NE, neutrophil; ALC, absolute lymphocyte count; RBC, red blood count; HB, hemoglobin; CRP, C reactive protein; β2-MG, beta-2 microglobulin; ApoA1, apolipoprotein A1; IL-6, interleukin-6; IL-10, interleukin-10, GLR, glucose-to-lymphocyte ratio.
