# Supplementary material for: Association between the glucose-to-lymphocyte ratio and prognosis in COVID-19 patients
Source: Front Cell Infect Microbiol. 2026 Sep 24;16:1881669. doi: 10.3389/fcimb.2026.1881669 (PMC13645524; doi:10.3389/fcimb.2026.1881669)
Supplement: Supplementary file 2 [file Table2.docx]

**Table S2. Univariate and multivariate analyses of different prognostic parameters for overall survival of 235 patients**

| **Variables** | **Univariate analysis for OS** | | **Multivariate analysis for OS** | | |
| --- | --- | --- | --- | --- | --- |
|  | ***P-value*** | **95%CI** | ***P-value*** | ***HR*** | **95%CI** |
| Age ≥ 60(years) | 0.279 | 47.279-53.222 | 0.234 | 2.240 | 0.594-8.453 |
| Gender (male) | 0.337 | 46.649-53.513 | 0.542 | 1.239 | 0.622-2.469 |
| CRP ≥ 52.23mg/L | <0.001 | 36.833-47.964 | 0.324 | 0.698 | 0.342-1.426 |
| IL-6 ≥ 38.7pg/ml | <0.001 | 26.422-41.605 | 0.714 | 0.853 | 0.364-1.999 |
| IL-10 ≥ 28.3pg/ml | <0.001 | 27.220-40.092 | 0.166 | 1.796 | 0.785-4.109 |
| WBC ≥ 14.9×10^9^/L | <0.001 | 26.359-42.650 | 0.348 | 0.402 | 0.060-2.702 |
| NE ≥ 13.3×10^9^/L | <0.001 | 24.798-42.128 | 0.958 | 0.947 | 0.123-7.309 |
| RBC < 3.57×10^9^/L | 0.035 | 36.628-45.501 | 0.670 | 0.830 | 0.351-1.960 |
| HB < 100g/L | 0.004 | 31.158-44.700 | 0.097 | 2.292 | 0.861-6.103 |
| PLT < 134×10^9^/L | 0.005 | 34.745-44.827 | 0.247 | 1.604 | 0.721-3.564 |
| Hypertension | 0.678 | 46.975-53.986 | 0.249 | 1.478 | 0.761-2.871 |
| Cardiopathy | 0.820 | 33.954-54.969 | 0.624 | 0.588 | 0.070-4.929 |
| Diabetes | 0.399 | 39.218-52.657 | 0.694 | 1.150 | 0.573-2.308 |
| Severe patients | <0.001 | 37.247-54.753 | <0.001 | 8.224 | 2.728-24.795 |
| β2-MG ≥ 8.89mg/L | <0.001 | 12.291-26.959 | 0.001 | 3.080 | 1.568-6.050 |
| ApoA1 < 0.68 g/L | <0.001 | 32.533-47.154 | 0.099 | 0.492 | 0.212-1.143 |
| GLR > 14.42 | <0.001 | 35.568-46.680 | 0.009 | 2.603 | 1.273-5.322 |

Abbreviations: WBC, white blood count; NE, neutrophil; RBC, red blood count; HB, hemoglobin; CRP, C reactive protein; β2-MG, beta-2 microglobulin; ApoA1, apolipoprotein A1; IL-6, interleukin-6; IL-10, interleukin-10, GLR, glucose-to-lymphocyte ratio.
